# M1- and M2-Type Macrophage Responses Are Predictive of Adverse Outcomes in Human Atherosclerosis

**DOI:** 10.3389/fimmu.2016.00275

**Published:** 2016-07-19

**Authors:** Monica de Gaetano, Daniel Crean, Mary Barry, Orina Belton

**Affiliations:** ^1^School of Biomolecular and Biomedical Science, UCD Conway Institute, University College Dublin, Dublin, Ireland; ^2^School of Veterinary Medicine, UCD Conway Institute, University College Dublin, Dublin, Ireland; ^3^St. Vincent’s University Hospital, Dublin, Ireland

**Keywords:** atherosclerosis, inflammation, macrophage phenotype, human plaque

## Abstract

Atherosclerosis is an inflammatory disease caused by endothelial injury, lipid deposition, and oxidative stress. This progressive disease can be converted into an acute clinical event by plaque rupture and thrombosis. In the context of atherosclerosis, the underlying cause of myocardial infarction and stroke, macrophages uniquely possess a dual functionality, regulating lipid accumulation and metabolism and sustaining the chronic inflammatory response, two of the most well-documented pathways associated with the pathogenesis of the disease. Macrophages are heterogeneous cell populations and it is hypothesized that, during the pathogenesis of atherosclerosis, macrophages in the developing plaque can switch from a pro-inflammatory (MΦ1) to an anti-inflammatory (MΦ2) phenotype and *vice versa*, depending on the microenvironment. The aim of this study was to identify changes in macrophage subpopulations in the progression of human atherosclerotic disease. Established atherosclerotic plaques from symptomatic and asymptomatic patients with existing coronary artery disease undergoing carotid endarterectomy were recruited to the study. Comprehensive histological and immunohistochemical analyses were performed to quantify the cellular content and macrophage subsets of atherosclerotic lesion. In parallel, expression of MΦ1 and MΦ2 macrophage markers were analyzed by real-time PCR and Western blot analysis. Gross analysis and histological staining demonstrated that symptomatic plaques presented greater hemorrhagic activity and the internal carotid was the most diseased segment, based on the predominant prevalence of fibrotic and necrotic tissue, calcifications, and hemorrhagic events. Immunohistochemical analysis showed that both MΦ1 and MΦ2 macrophages are present in human plaques. However, MΦ2 macrophages are localized to more stable locations within the lesion. Importantly, gene and protein expression analysis of MΦ1/MΦ2 markers evidenced that MΦ1 markers and Th1-associated cytokines are highly expressed in symptomatic plaques, whereas expression of the MΦ2 markers, mannose receptor (MR), and CD163 and Th2 cytokines are inversely related with disease progression. These data increase the understanding of atherosclerosis development, identifying the cellular content of lesions during disease progression, and characterizing macrophage subpopulation within human atherosclerotic plaques.

## Introduction

Atherosclerosis is a progressive disease characterized by the accumulation of lipids and fibrous elements in large- and medium-sized arteries, driven by several genetic and environmental factors (1–4). A large number of risk factors for the disease have been identified, such as elevated low-density lipoprotein (LDL) cholesterol, hypertension, obesity, and type-2 diabetes. Atherosclerotic plaques are initiated by the accumulation of LDL and the recruitment of inflammatory cells in particular monocytes, at sites of endothelial dysfunction (5). Monocyte migration and their differentiation into macrophages by exposure to macrophage colony-stimulating factor (M-CSF) is followed by subsequent foam cell formation, arising from macrophage receptor-mediated uptake of oxidized LDL (ox-LDL), and a proliferative response of smooth muscle cells (6). Plaque rupture triggers ~76% of all fatal coronary thrombi (7). Established atherosclerotic patients are classified as either “symptomatic,” where the patient has experienced previous ischemic events but without any cardiovascular disease (CVD) diagnosis; or “asymptomatic,” where a patient has no history of ischemic events or CVD. Among patients with symptomatic carotid artery stenosis, surgical removal of the advanced plaque, by carotid endarterectomy (CEA), is still one of the most performed surgical procedures, due to its associated lower risk of peri-procedural stroke or death, in comparison with other types of intervention, such as carotid artery stenting (8). Although conventional therapies, such as statins, which decrease plasma LDL levels, and antihypertensives control the risk factors for the disease, there are currently no available therapies that effectively regress pre-established plaques (9). The implications of this are important as most people present clinically with established disease and the therapeutic goal would be to reverse the established lesion. While most of the cellular and molecular mechanisms in the development of atherosclerotic lesions have been identified, there is limited information on those pathways and processes governing regression. Indeed, identifying novel cellular processes associated with atheroprotection or those which regulate regression is critical to the identification of novel therapeutic targets.

Macrophages are phagocytic cells derived from bone marrow precursors and monocytes. They are essential for the maintenance and defense of host tissues, as they sense and engulf cellular debris and pathogens. They are also able to stimulate a pro-inflammatory response by stimulating lymphocytes and other immune cells to respond to the pathogen (10). One of the most critical functions of macrophages, in the context of atherosclerosis, is lipid uptake and deposition and the subsequent disease progression. ox-LDL is recognized by macrophage scavenger receptors (SRs), predominantly CD36 and CD68. SRs mediate the uptake of highly ox-LDL by macrophages, leading to an increase in intracellular lipid accumulation and consequently, foam cell formation (11). Intracellular cholesterol is then metabolized and transported to exogenous acceptors, such as high-density lipoprotein, through efflux proteins, such as ATP-binding cassette (ABC) transporters, ABCA1 and ABCG1 (12–14).

We have previously shown that dietary administration of conjugated linoleic acid (CLA), a family of naturally occurring geometric dienoic isomers of the ω6 essential fatty acid, linoleic acid (15) induces regression of pre-established atherosclerosis in the apoE mouse model of atherosclerosis despite a continuing high cholesterol challenge (16). Furthermore, we have recently identified the monocyte/macrophage as the cellular target through which CLA mediates this profound effect (17, 18). Of relevance to this present study, we have shown, in both *in vivo* and *in vitro*, that CLA primes macrophages toward an anti-inflammatory macrophage phenotype and limits macrophage accumulation and foam cell formation by modulating the expression of both SRs and efflux proteins, ultimately, altering cholesterol trafficking in regression of pre-established atherosclerosis (19, 20). This suggests that altering the macrophage phenotype may be of therapeutic importance in established disease. However, currently there is limited information regarding the localization and function of macrophage subpopulations in the progression of atherosclerosis particularly in progression from asymptomatic to symptomatic disease.

Over the last decade, the heterogeneity of macrophage populations has been extensively documented (21). Two types of macrophages have been identified based on the exposure to different cytokine environments. The “classically activated,” MΦ1, pro-atherogenic macrophages are primed by exposure to T helper (Th)-1 cytokines, such as IFNγ and IL-1β; while “alternative,” MΦ2, anti-inflammatory macrophages are induced as a result of exposure to Th2 cytokines, such as IL-4 and IL-3 (22). Macrophages are phenotypically plastic cells, in that they can switch from MΦ1 to MΦ2 state and *vice versa* upon specific signals (23, 24). Due to their enhanced phagocytic activity in early plaque development, the anti-inflammatory properties of MΦ2 macrophages, in comparison to MΦ1 pro-inflammatory, macrophages have been highlighted (24). It is suggested that atherosclerosis may be caused not only by a sustained pro-inflammatory reaction but also by impaired anti-inflammatory, pro-resolving mechanisms (21). For example, it has been shown that the presence of macrophages in human atherosclerotic plaques are indicative of a chronic inflammatory reaction (25). The majority of previous studies identifying macrophage subsets in human atherosclerosis have compared atherosclerotic plaques with normal vessels and while they have confirmed the heterogeneity of the macrophage subpopulations (26), data on macrophage plasticity in the context of human atherosclerotic disease progression is limited. Shaikh et al. showed using immunohistochemistry analysis, that plaques from patients with recently symptomatic carotid disease have a predominance of M1 macrophages and higher lipid content compared to femoral plaques, consistent with a more unstable plaque (27). Of relevance to this study is that the relationship between macrophage polarization and the vulnerability of human atherosclerotic plaques has been investigated. Macrophage-rich areas were identified by CD68^+^ immunostaining and showed that plaques from symptomatic patients had a greater concentration of macrophages specifically M1 macrophages. By contrast, increased expression of markers associated with alternative M2 macrophage differentiation was observed in plaques from the asymptomatic group (28).

The focus of this study was to comprehensively analyze macrophage subsets in human atherosclerosis progression by comparing cellular content and macrophage populations in plaques from asymptomatic and symptomatic patients. To this end, several macrophage markers were analyzed. Expression of the ox-LDL SRs, CD36 and CD68 was examined, as although both receptors mediate uptake of modified lipoprotein, they have different cellular localizations. CD36 is expressed on the plasma membrane and has previously been used to identify both MΦ1 and MΦ2 macrophage subsets, while CD68 is located on the lysosomal surface (25, 29). To examine the expression of cytokines known to induce an MΦ1 phenotype, we performed mRNA expression analysis of Th1-cytokines (TNFα, IL-1β, IL-6, IL-8, IL-12p40, and IL-12p35) and of Th1-chemokines (MCP-1 and CXCL10) (30). In addition, MR, CD163 and Dectin-1 were used as established MΦ2 markers (24, 27, 31, 32). MR is a type-I membrane receptor protein that is found on the macrophage surface, where it mediates the endocytosis of glycoproteins by binding high-mannose structures on the surface of potentially pathogenic viruses, fungi, and bacteria, enabling them to be neutralized by phagocytic engulfment. During inflammation, MR is crucial for rapid clearance of several mannose-bearing serum glycoproteins but does not regulate the initiation of inflammation (33, 34). The hemoglobin–heptaglobin SR, also known as CD163, is a macrophage-specific protein and a hallmark of the macrophage switch to MΦ2 phenotype (35). Dectin-1 is a β-glucan receptor expressed on leukocytes, mediating all the immunomodulatory effects of carbohydrates, including the β-glucan-dependent binding of zymosan, a yeast-derived particle composed principally of polysaccharides (32). Moreover, Dectin-1 has been recently associated with the production of high levels of IL-10 but low levels of IL-12p40, thus, shifting the macrophage phenotype toward an M2b or “regulatory” macrophage (36). Furthermore, we also examined expression of cytokines (IL-10, IL-4, and IL-13) and chemokines (CCL22 and CCL18) which are known to induce an MΦ2 phenotype (37).

There remains a lack of consensus on whether the cholesterol efflux proteins, ABCA1 and ABCG1, and the SR, SRA-1, are associated with an MΦ1 or MΦ2 phenotype (24, 38). ABCA1 exports cholesterol to lipid-free apolipoproteins (apo), involving an initial interaction of apoAI with lipid rafts located on the macrophage surface, while ABCG1 effluxes cholesterol to phospholipid-containing acceptors, such as HDL, in a lipid raft-independent manner (27, 39). The canonical route for ox-LDL to enter the cell is *via* SR-mediated uptake and it has been extensively shown that the pro-inflammatory cytokine, IFN-γ, increases the expression of the SR SRA-1, in both THP-1- and HBPMC-derived macrophages (40).

Thus, the expression of ABCA1, ABCG1, and SRA-1 in asymptomatic and symptomatic atherosclerotic plaques was also examined to identify if they are altered in disease progression and to establish if they are predominantly associated with an MΦ1 or MΦ2 phenotype.

Therefore, the overall aim of the present study was to elucidate the differential macrophage populations in atherosclerosis progression, through the analysis of cellular content and the relative distribution of MΦ1 and MΦ2 macrophage populations in human asymptomatic and symptomatic plaques.

## Materials and Methods

### Patients

#### Tissue Specimen Preparation

**Figure 1:**
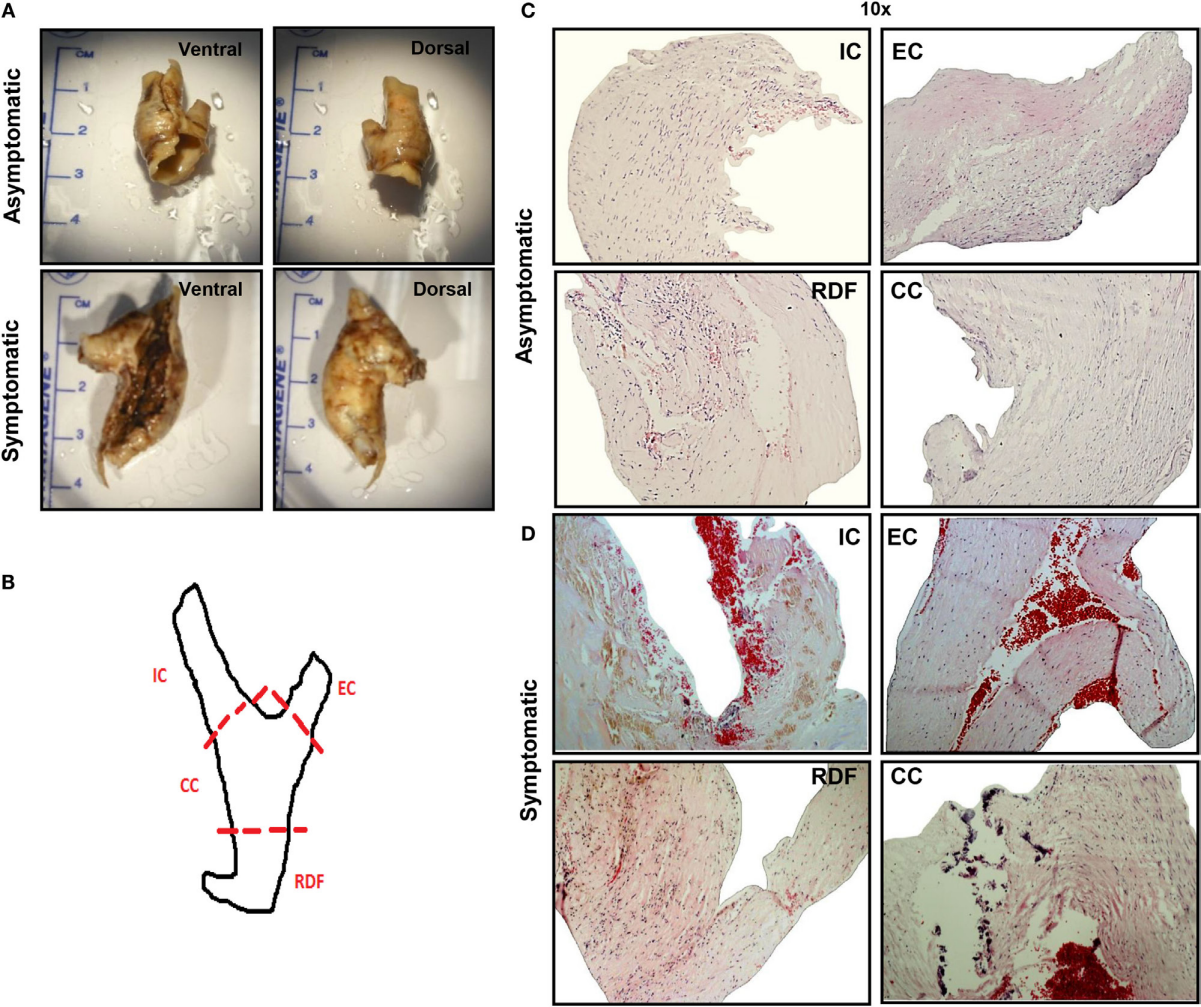
**Comprehensive analysis of asymptomatic vs. symptomatic plaque**. **(A)** *Gross analysis*. After collection, atherosclerotic plaque tissues were analyzed at gross level in order to detect evident signs of hemorrhage or calcification and to measure plaque size. Generally, asymptomatic patients presented smaller lesions with less evident signs of hemorrhage or calcification, compared to symptomatic patients. Here, representative images for each group of patients are displayed. **(B)** *Dissection*. Schematic of carotid atherosclerotic plaque dissection where dotted lines indicate the points of dissection, and the resulting sections are labeled as follows: common carotid (CC), the main arterial element and origin of disease; internal carotid (IC), the larger bifurcation from the CC; external carotid (EC), the smaller bifurcation from the CC and the relatively disease-free (RDF) component, least diseased region of the artery due to its peripheral location. **(C,D)** *H&E staining*. After dissection, H&E staining was performed on 6 μm sections. A representative comparison between **(C)** asymptomatic and **(D)** symptomatic plaque is shown. Hemorrhagic signs, calcification, and necrotic depositions are clearly visible in all dissected sections from the symptomatic plaques, with the exception of RDF. Pictures were captured using TLM (10× magnification). Representative images of *N* = 3 independent staining are displayed here.

The study was approved by the Ethics Committee of St. Vincent’s University Hospital, Dublin and in accordance with International guidelines and Helsinki Declaration principles. All patients gave written informed consent. Patients with clinical and angiographic evidence of atherosclerosis undergoing revascularization surgery were recruited to the study. Immediately following CEA, human carotid atherosclerotic plaques specimens were either fixed in formalin for 24 h, and stored at room temperature for subsequent immunohistochemical analysis, or placed in “RNA later” solution, and stored at −80°C for RNA and protein analysis. Photographs were taken of whole plaques prior to dissection, in order to document the dorsal and ventral orientation of the specimen. Each plaque specimen was then dissected into four distinct sections: common carotid (CC), internal carotid (IC), external carotid (EC), and relatively disease free (RDF), as shown in Figure 1A. Each plaque section was individually placed in plastic cassettes and processed for 9 h through a cycle of alcoholic and organic solvents, ending with a wax-embedding step, using an automated instrument, before being manually embedded in paraffin wax, as described in detail in Table S1 in Supplementary Material.

Paraffin-embedded sections were then cut into slices of 6 μm of thickness. This process was used to obtain serial sections of each sample to be analyzed. The paraffin sections were mounted on polylysine-coated slides in a water bath at 37°C. The slides were then placed in the histology oven for 1 h to secure adhesion of tissue to the slide.

#### H&E Staining

After deparaffinization in xylene and subsequent rehydration in graded alcohol series (Table S2 in Supplementary Material), Mayer’s Hematoxylin (HX) (Sigma Aldrich, Ireland) was applied to each tissue section for 5 min, in order to stain nuclei. After washing, acidic ethanol (99 ml of 70% ethanol and 1 ml of Hydrochloric acid) was added for 5 s to de-stain. Eosin (Merck, Ireland) was then added to each section for 5 min to visualize the cytoplasm and the extracellular matrix of the tissue. Slides were then washed and dehydrated as described in Table S3 in Supplementary Material. Mounted slides were allowed to dry overnight before transmission light microscopy (TLM) visualization, using Nikon Eclipse 80i and the NIS-Elements BR software.

#### IHC Staining Procedure

After deparaffinization in xylene and subsequent rehydration in graded alcohol series, endogenous peroxidases were quenched using 3% H_2_O_2_ (Merck, Ireland) in 30% methanol (Sigma Aldrich, Dublin, Ireland) for 30 min. After washing, non-specific antibody binding was prevented by blocking with serum in which the antibody was raised for 1 h at room temperature. Following washes, antibodies against human CD68 (1:100), SMA (1:100) (both from DAKO, Glostrup Denmark) and MR (1:50) (Sigma Aldrich, Dublin, Ireland) were incubated overnight at 4°C. After washing, secondary biotinylated horseradish peroxidase (HRP) Ab was incubated for 1 h at room temperature. Once washed, VECTASTAIN Universal Elite ABC solution (Vector Laboratories), an Avidin Biotinylated peroxidase Complex, was added for 30 min. Following washing steps, 3,3′-Diaminobenzidine (DAB, Sigma Aldrich, Dublin, Ireland) brownie chromogen was placed for 2–5 min as peroxidase substrate to localize peroxidase in tissue. Mayer’s HX was used as counterstain for 90 s. After washing, dehydration steps followed. Mounted slides were allowed to dry before TLM visualization or before scanning with Aperio ScanScope XT digital scanner. Negative controls were obtained omitting addition of primary Ab, secondary Ab, DAB or HX, as shown in Figure S1 in Supplementary Material. All subsequent analysis and quantification was performed by a trained researcher blinded to the symptomatic status of the patients.

#### IHC Staining Visualization and Quantification

Stained slides were visualized using the Aperio ScanScope XT digital scanner and its software Aperio ScanScope Console. Images were then stored on the “Spectrum” platform for analysis. IHC staining was quantified using the Aperio “ImageScope” software and its required algorithms. The first algorithm used was “Colour Deconvolution,” which calibrated the immunostaining for DAB and HX of each plaque sample stained, giving a basal level of the target protein expression.

Nuclear analysis was then carried out to, first, establish the target protein expression per cell and, second, as a total per tissue section. “V9 Nuclear algorithm,” using the baseline values previously produced by Colour Deconvolution, quantified the intensity of DAB staining (protein) in each HX (nuclei) stained cell and assigned a mark-up image that was more easily quantifiable. The mark-up images applied different arbitrary colors to corresponding levels of protein expression. Counting of cells, for each different color-coded level of protein expression, was elaborated by the software, as we have previously described (41).

For each tissue section, every 10 slides cut (composing a “series” progressively numbered), one slide was stained using the same primary antibody, in order to have three “series” of slides (60 μm apart) throughout the tissue; thus, representative of the whole specimen analyzed (for example, CD68 staining was performed on the 1st, 11th, and 21st slide; MR on the 2nd, 12th, and 22th; SMA on the 3rd, 13th, and 23rd). An average quantification of the three series for each marker was then calculated.

#### Gene Expression Analysis

For gene expression experiments, specimens stabilized in RNA later solution were used to extract total RNA from tissues using Trizol (Invitrogen, Ireland). Reverse transcription was carried out on 1 μg of total RNA using Superscript™ III Reverse Transcriptase (Invitrogen, Ireland) according to the manufacturers’ instructions. Relative gene expression quantification by real-time PCR (RT-PCR) was performed on an ABI Prism 7900HT Sequence Detection System (Applied Biosystem Inc., UK). MR, SRA-1, and ABCG1 expression were examined using specific Taqman assays (Applied Biosystems Inc., UK), while TNFα, IL-1β, IL-6, IL-8, IL-12p40, IL-12p35, CXCL10, MCP-1, Dectin-1, CCL22, CCL18, IL-10, IL-4, IL-13, ABCA1, CD14, CD36, CD68, and CD163 target genes were measured using specific Syber green assays (Eurofins, MWG Operon, Germany) and Ct values were normalized to 18 s ribosomal RNA (Table S4 in Supplementary Material).

#### Western Blot Analysis

Total tissue protein lysates were separated using 4–20% SDS-polyacrylamide gel and transferred to nitrocellulose membranes. Membranes were blocked with 3% BSA in TBS-T at room temperature prior to overnight incubation with primary antibodies at 4°C with gentle shaking. Detection was performed using a HRP-conjugated secondary antibody incubated for 1 h at room temperature and ECL detection system. Mouse-monoclonal anti-human β-actin (1:1000); goat-polyclonal anti-human CD68 (1:500); rabbit-polyclonal anti-human MR (1:500); and ABCA1 (1:1000) were used. β-actin, CD68, and MR were purchased from Santa Cruz Biotechnology, CA, USA. ABCA1 was purchased from Novus, CO, USA. The following HRP-conjugated secondary antibodies were as follows: polyclonal anti-rabbit secondary antibody (Cell Signaling Technology, MA, USA); polyclonal anti-mouse secondary antibody (Santa Cruz Biotechnology, CA, USA).

### Statistical Analysis

The data were analyzed by parametric unpaired Student’s *t*-test and analysis of variance (ANOVA). The student *t*-test was used to compare the mean of two data sets. ANOVA was used to examine any overall differences between groups. Results are expressed as mean ± SEM. Experimental points were performed in triplicate with a minimum of three independent experiments. A value of *p* < 0.05 or smaller was considered statistically significant.

## Results

### Patients

Eighty-seven patients undergoing CEA were recruited to this study, three of which had bilateral lesions. The “symptomatic status” of the patients at the time of surgery was available for 80 patients based on their clinical records. Thirty-four out of 80 patients (42.5%) were classified as “symptomatic,” while 46 out of 80 (57.5%) were defined as “asymptomatic.” The average age of all patients was 68 ± 3.6. However, the average age was slightly higher in the symptomatic (71 ± 7.8 years) group than in the asymptomatic group (66 ± 7.6 years) (Table 1). The characteristics of the patients analyzed in this study for IHC or mRNA/protein analyzes displayed a similar trend as the one described above (Table 2).

**Table 1:** **Population profile**.

	CEA frequency relative to gender, *N* (%)	CEA frequency relative to symptomatic status, *N* (%)	Average age	Range
*All patients*	*80 (100)*	*80 (100)*	*68.0*	*47–83*
Symptomatic	34 (42.4)	34 (100)	70.2	51–83
Asymptomatic	46 (57.5)	46 (100)	65.6	47–83
*Males*	*60 (100)*	*60 (75)*	*67.1*	*47–83*
Symptomatic	24 (40)	24 (71)	67.9	51–83
Asymptomatic	36 (60)	36 (78)	65.8	47–83
*Females*	*20 (100)*	*20 (15)*	*69.9*	*60–83*
Symptomatic	10 (50)	10 (29)	74.6	65–83
Asymptomatic	10 (50)	10 (22)	65.2	60–70

*Characteristics (gender, age, and symptomatic status) of the 80 patients recruited to the study that underwent CEA. The average age is higher in symptomatic vs. asymptomatic, in female vs. male and in symptomatic females vs. symptomatic males*.

**Table 2:** **Characteristics of patients analyzed in the study**.

	CEA frequency relative to gender, *N* (%)	CEA frequency relative to symptomatic status, *N* (%)	Average age	Range
*All patients*	*18 (100)*	*18 (100)*	*64.6*	*41–83*
Symptomatic	9 (50)	9 (100)	70.4	41–83
Asymptomatic	9 (50)	9 (100)	58.7	43–71
*Males*	*15 (100)*	*15 (83)*	*63.0*	*41–83*
Symptomatic	7 (47)	7 (78)	68.1	41–83
Asymptomatic	8 (53)	8 (89)	58.5	43–71
*Females*	*3 (100)*	*3 (17)*	*72.6*	*61–79*
Symptomatic	2 (67)	2 (22)	78.5	78–79
Asymptomatic	1 (37)	1 (11)	61	–

*Plaques from 18 patients that were analyzed for either H&E/IHC analysis or for RNA/protein expression. Table shows gender, age, and symptomatic status. The average age is higher in symptomatic vs. asymptomatic, in female vs. males and in symptomatic females vs. symptomatic male. The plaques from the patients analyzed in this study are representative of the whole population recruited as described in Table 1*.

### Atherosclerotic Plaque Gross Analysis

Prior to dissection of the plaque samples, the dorsal and ventral surface of each specimen was photographed to detect gross calcification and identify evidence of hemorrhagic plaques. Figure 1A shows representative plaques from symptomatic and asymptomatic patients. All plaques analyzed from asymptomatic patients displayed no evidence of calcification and very little or no hemorrhagic spots. By contrast, all symptomatic plaques had extensive hemorrhaging and had partial or severe calcification. The average size of asymptomatic plaques was smaller than that of symptomatic (2.8 ± 0.4 vs. 3.8 ± 0.2 cm), which is in keeping with the assumption that symptomatic patients present with more advanced and/or complicated lesions. Therefore, gross analysis of plaque specimens confirmed that comparison of asymptomatic and symptomatic plaques is a valid model of disease progression.

### H&E Analysis of Asymptomatic and Symptomatic Plaques

H&E staining was performed on sections from all plaques. Figure 1 shows a representative overview of the cellular composition of plaques from both asymptomatic and symptomatic patients. Dotted lines in the diagram indicate how the surgically removed plaque specimen was dissected into “CC,” the area where vessel bifurcation originates, which is also the most prone region for plaque development, “IC,” the main arterial element and site of origin of disease, at the interphase with CC; “EC,” the smaller bifurcation from the CC and, finally, the “RDF” component, least diseased region, due to its peripheral location that was used as a control (Figure 1B). Asymptomatic plaques were homogeneous in terms of cellular content and absence of hemorrhagic signs (platelets and red blood cells stained in red), except for evidence of early hemorrhagic activity in the IC and EC sections, which are located at the site of bifurcation of the vessel, representing the more atheroprone regions (Figure 1C). Conversely, plaques from symptomatic patients showed greater hemorrhagic activity. Overall, IC was considered the most diseased portion on the basis of the predominant prevalence of calcifications, fibrotic, and necrotic tissue and hemorrhagic events, therefore, it was used in subsequent immunohistochemistry analysis for comparisons with RDF and for comparison between symptomatic vs. asymptomatic lesions (Figure 1D).

There were obvious differences between the developing core and the necrotic core in asymptomatic and symptomatic plaques. Indeed, early to intermediate stages of the disease are characterized by the fibrotic tissue that starts to replace cells, such as macrophages, foam cells, and SMCs, whereas at later stages, the first visible clinical sign of the disease, known as “fatty-streak” is identified by cholesterol crystal deposits that are highly concentrated in that region (Figure S2 in Supplementary Material). Cholesterol precipitates in the extra cellular matrix as a consequence of the release on the outer space of the lipid content of dead foam cells due to necrotic processes.

### Cellular Composition of Symptomatic and Asymptomatic Plaques

**Figure 2:**
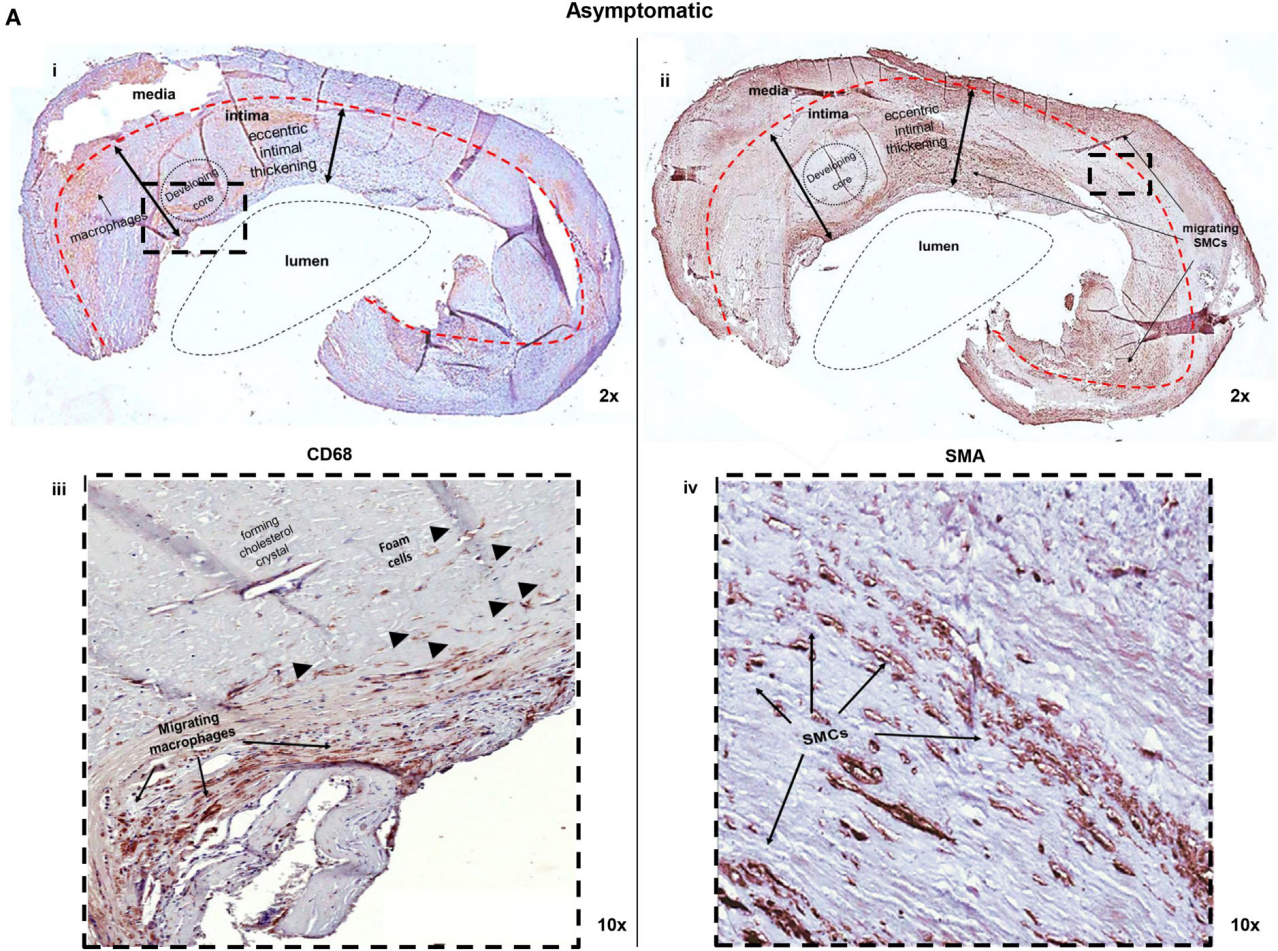

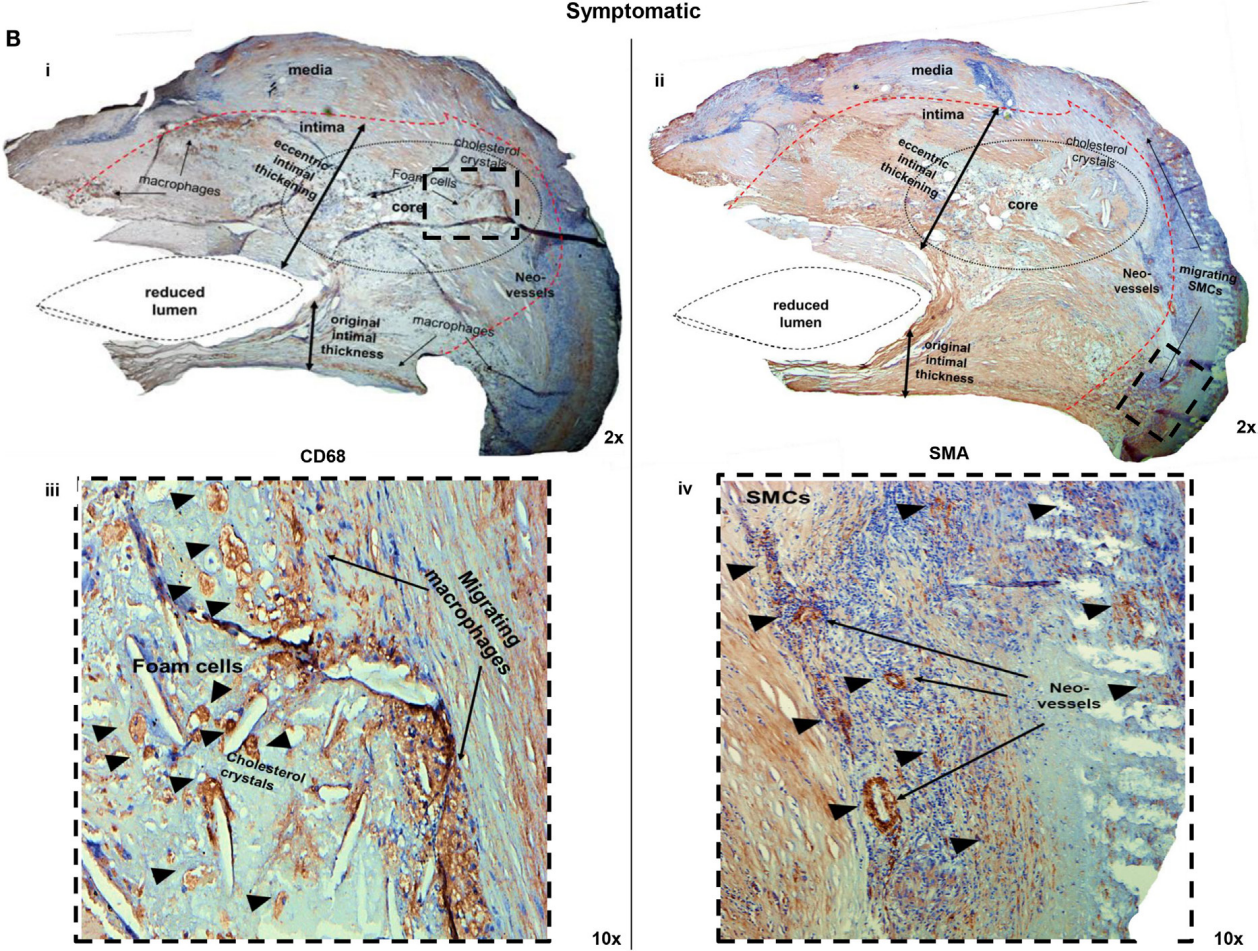
**Macrophage/foam cell and SMCs content is decreased in (A) asymptomatic and increased in (B) symptomatic plaques**. **(A)** Low (2×) (i,ii) and high (10×) (iii,iv) magnifications of the IC portion of a plaque from an asymptomatic patient, where the central lumen, intimal side, and media layer of the plaque are visible. Sections were stained with an anti-CD68 Ab (i,iii) to identify macrophage accumulation. Macrophages were identified migrating toward the developing core with little detection of foam cells *(head arrows)*. Sections were also stained with an anti-SMA Ab (ii,iv). SMCs *(arrows)* display migratory activity, moving from media to intimal layer. **(B)** Low (2×) (i,ii) and high (10×) (iii,iv) magnifications, of the IC portion of a plaque from a symptomatic patient, identifying a reduced lumen compared to asymptomatic plaques (i), an intimal side and a media layer of the plaque. The intimal thickness is also increased compared with asymptomatic plaques (i,iii). Macrophages *(arrows)*, detected with an anti-CD68 Ab, are evident in the necrotic core and foam cell accumulation *(head arrows)* are predominant. (ii,iv) Extensive SMCs activity *(head arrows)*, detected with an anti-SMA Ab, is evident primarily in the intima layer. Pictures were captured using TLM (2× and 10× magnifications). Representative images of *N* = 3 independent staining are displayed here.

H&E staining was employed to highlight the general structure of the lesions (hemorrhage, calcification, connective tissue, and fibrous/lipid burden). However, identification and localization of specific cell types, such as macrophages, macrophage-derived foam cells, and SMCs, are best achieved using immunohistochemistry analysis, as shown in Figure 2. Combined analysis of the two staining procedures allows to definitively establishing the orientation of the plaque, identifying the luminal side and the intimal and medial layers. Macrophages and foam cells were identified using a human anti-CD68 Ab, as a specific marker (Figure 2A, i,iii and Figure 2B, i,iii). Macrophages were found throughout the plaque sections, while foam cells preferentially localized in the developing or necrotic core of both asymptomatic and symptomatic plaques. In addition, vascular SMCs, determined at H&E level by their characteristic elongated nucleus, were confirmed by IHC using a human anti-SMA Ab, as a specific marker. These cells localize to the medial side of the plaque (Figure 2A, ii,iv and Figure 2B, ii,iv).

In particular, as disease progress, the lumen of the vessel is reduced and the intimal thickness is increased, according to the necrotic core development. Macrophages migrate toward the core, phagocytizing lipids and transforming into foam cells, reaching the forming atheroma, where necrotic processes occur. Here, necrotic foam cells are recognized by the presence of cholesterol crystals, a hallmark of the lipids released in the extra cellular matrix, promoting an inflammatory status (Figure 2B, iii). In symptomatic lesion, extensive SMCs migratory activity is also displayed, moving from media to intima layer, forming the structure of neo-vessels during neo-angiogenetic processes (Figure 2B, iv). Overall, IHC analysis highlights that both cell types are active within atherosclerosis.

### Localization and Quantification of MΦ1 and MΦ2 Macrophage in Lesions

**Figure 3:**
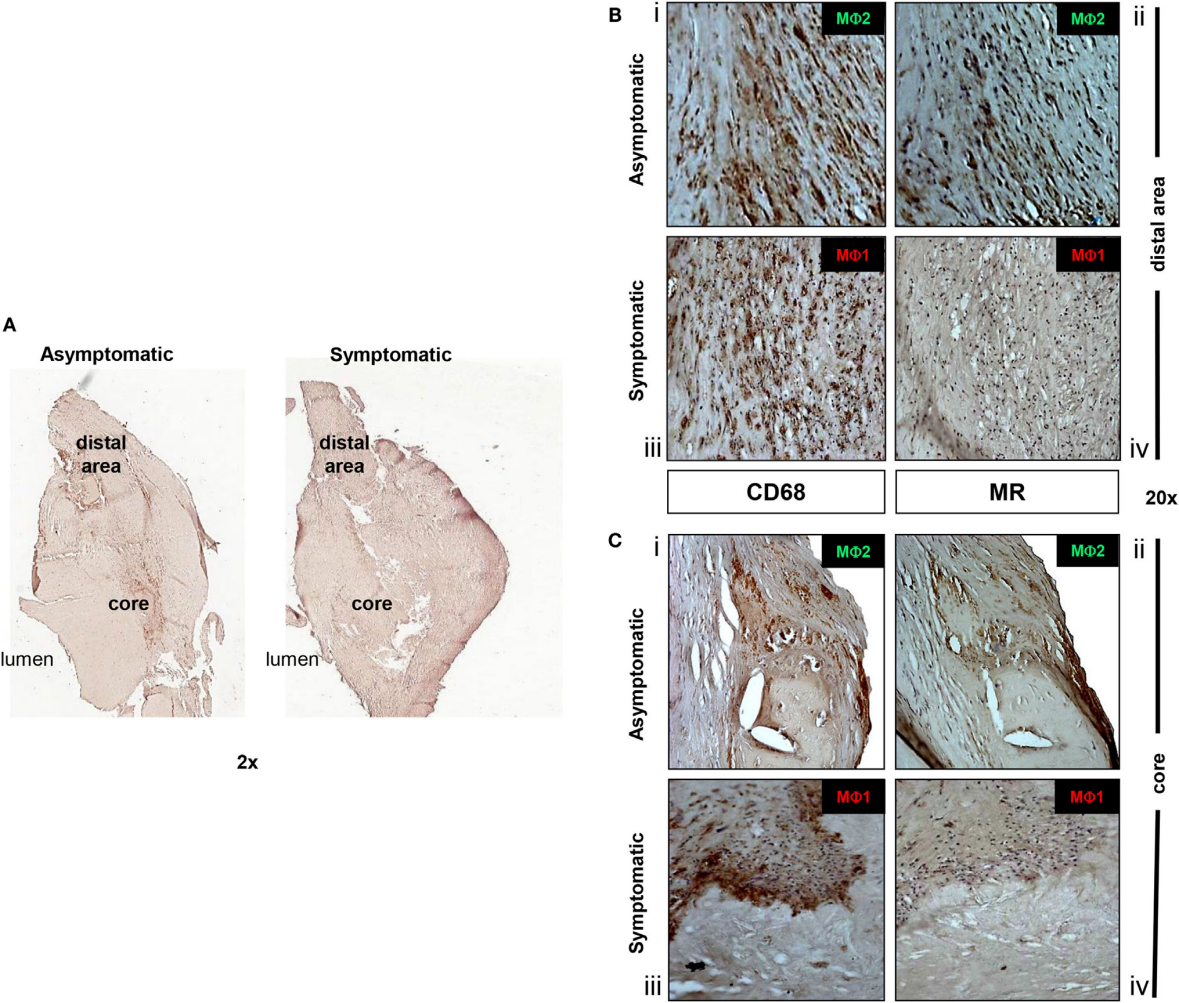
**Anti-inflammatory MΦ2 macrophages are decreased in symptomatic plaques**. **(A)** In both asymptomatic and symptomatic lesions, a central area, corresponding to the lesion initiation site (*core*) and a peripheral region (*distal area*) of the plaque were identified. In the “distal area” of asymptomatic patients, MΦ2 (CD68^+^/MR^+^) are predominant **(B)** (i,ii). Although still present in peripheral areas, MΦ2 populations are also located in the developing core of the asymptomatic plaques **(C)** (i,ii). Conversely, symptomatic lesions are characterized by the predominance of MΦ1 macrophages (CD68^+^/MR^-^), in both the distal and developed core **(B)** (iii,iv) and **(C)** (iii,iv). Pictures were captured using TLM (2 and 20× magnifications). Representative images of *N* = 3 independent staining are shown.

For the next set of experiments, we focused on the analysis of macrophage subpopulations, the “classically activated” MΦ1 pro-inflammatory macrophages and the “alternative” MΦ2 anti-inflammatory macrophage to gain insight into macrophage plasticity during atherosclerotic disease progression. Their localization, differentiation, and distribution relative to one another were initially examined by IHC staining for both CD68, a macrophage marker previously used to identify macrophages in human atherosclerotic plaques (23, 25, 42), which we used to identify the total macrophage population in each plaque, and for MR, specifically used to discriminate between MΦ1 (CD68^+^/MR^-^) and MΦ2 (CD68^+^/MR^+^) macrophage subpopulations (24). First, a peripheral region, named “distal area,” characterized by the original thickness of the vessel prior to the development of the lesion, was identified. Subsequently, a developing or established necrotic region (core) was found in an area characterized by eccentric thickening of the vessel (Figure 3A). IHC analysis of asymptomatic plaques identified that macrophages were only found abundantly in the lipid core of the plaque and rarely in the intimal space (Figure 3B, i,ii and Figure 3C, i,ii). By contrast, in symptomatic plaques, macrophage accumulation was evident in both the developed lipid core and the distal area of the diseased portion of the plaque (Figure 3B, iii,iv and Figure 3C, iii,iv). Our data based on the expression of MR, relative to the presence of CD68 marker, concurs with that of others, show that there is more than one population of macrophage present in atherosclerotic lesions. Furthermore, we showed that MR^+^ macrophages in asymptomatic plaques were localized to more stable distal areas and were also modestly present around the developing core, compared to MΦ1 macrophages; while in symptomatic plaques, there was a decrease in MR expression specifically in the core but also to some extent in the distal area, as evidenced in macrophage localization analysis of symptomatic plaques. The specificity of this staining was verified by the use of negative controls (Figure S1 in Supplementary Material).

### Differential Expression of MΦ1 and MΦ2 Macrophage Markers in Symptomatic and Asymptomatic Atherosclerotic Patients

Several markers predominantly expressed by specific subsets of macrophages have now been identified (43). The aim of the next series of experiments was to quantify differential expression of established MΦ1/MΦ2 markers and Th1/Th2 cytokines and chemokines in human atherosclerotic plaque and to address if there are changes in gene and/or protein expression associated with disease progression.

In addition, due to the fact that a major function of macrophages in the context of atherosclerotic disease progression is in cholesterol trafficking, we examined the expression of the SR SRA-1 and the ABC-transporter proteins ABCA1 and ABCG1 to identify if they are associated with either an MΦ1 or MΦ2 phenotype and if they are altered in human disease progression.

**Figure 4:**
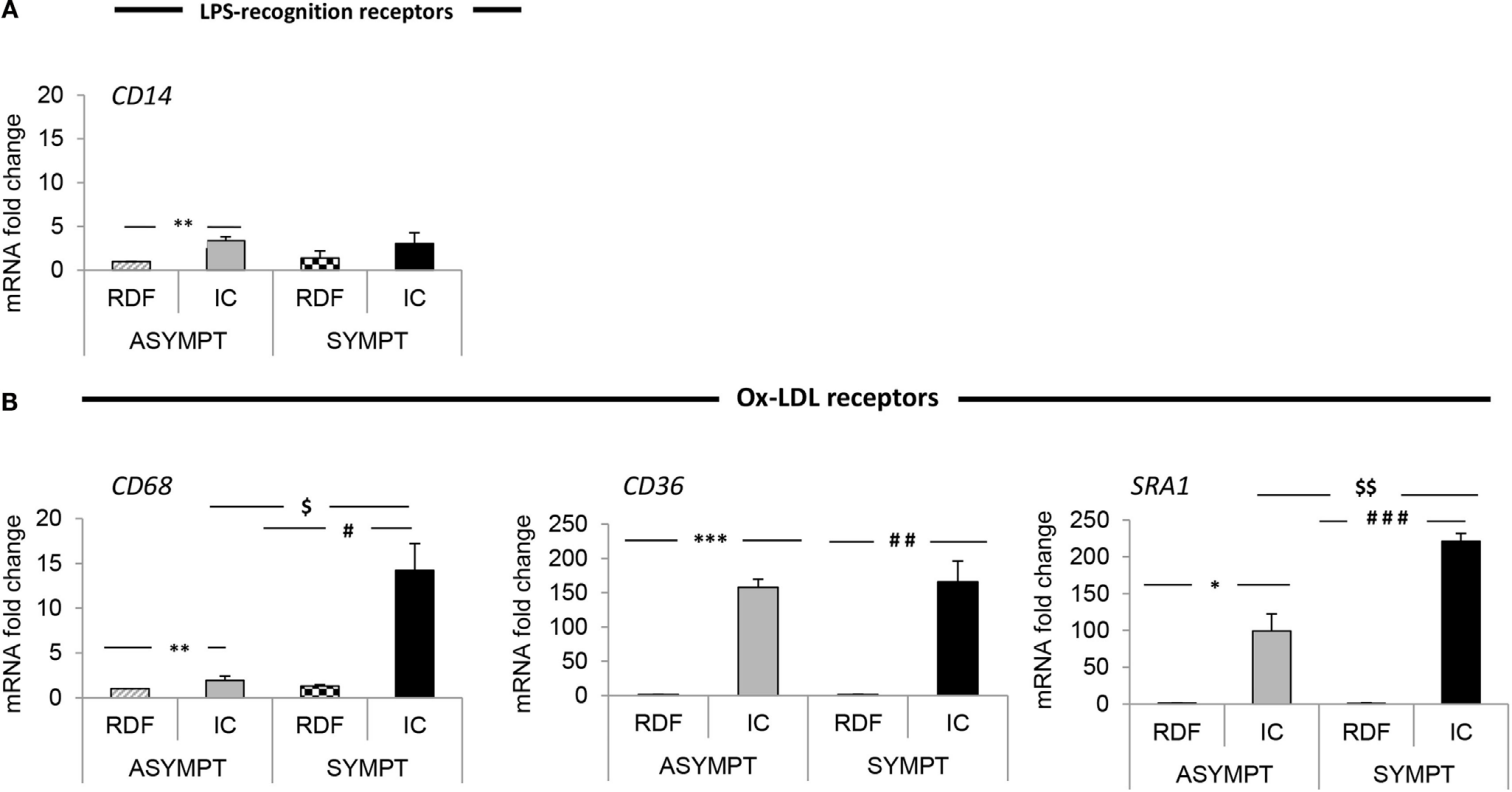

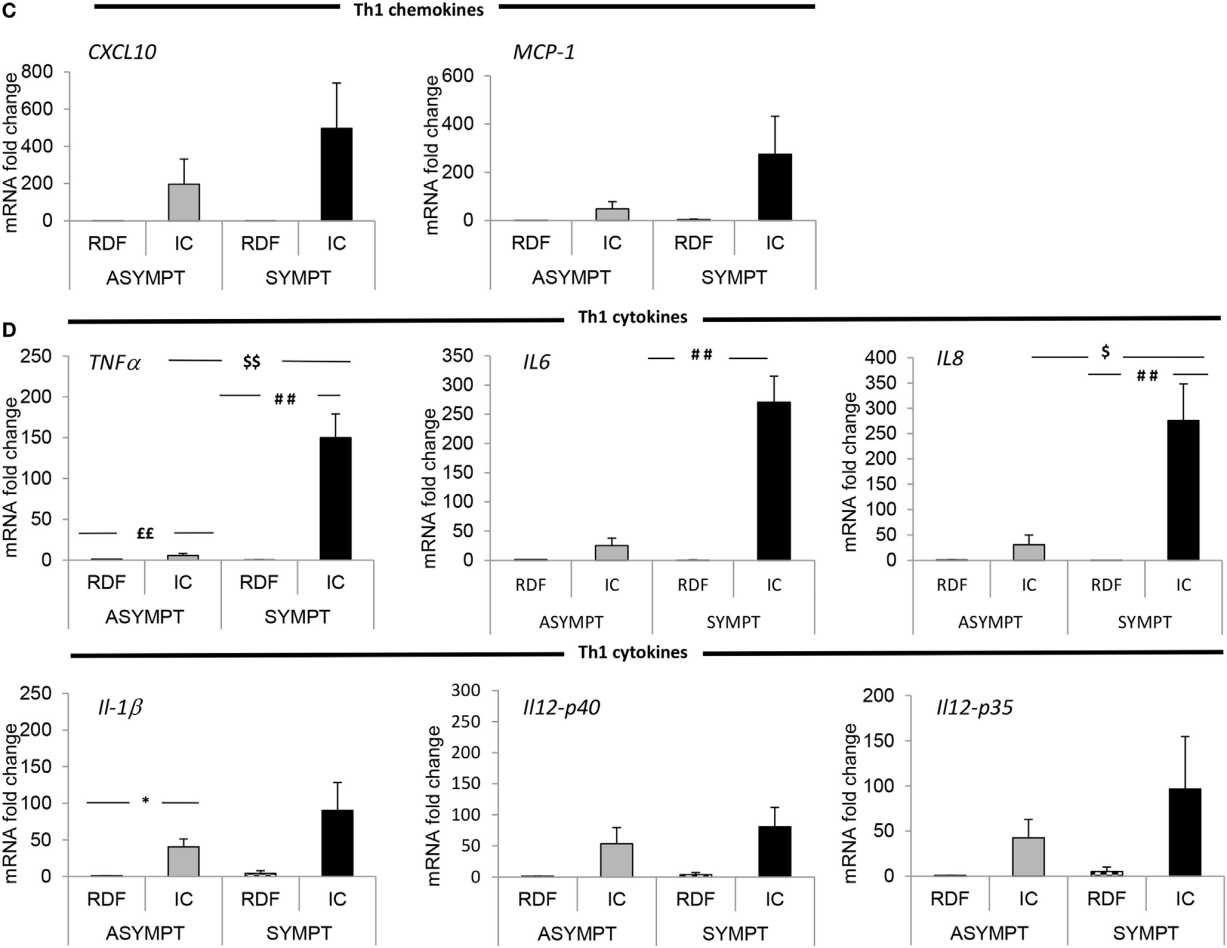

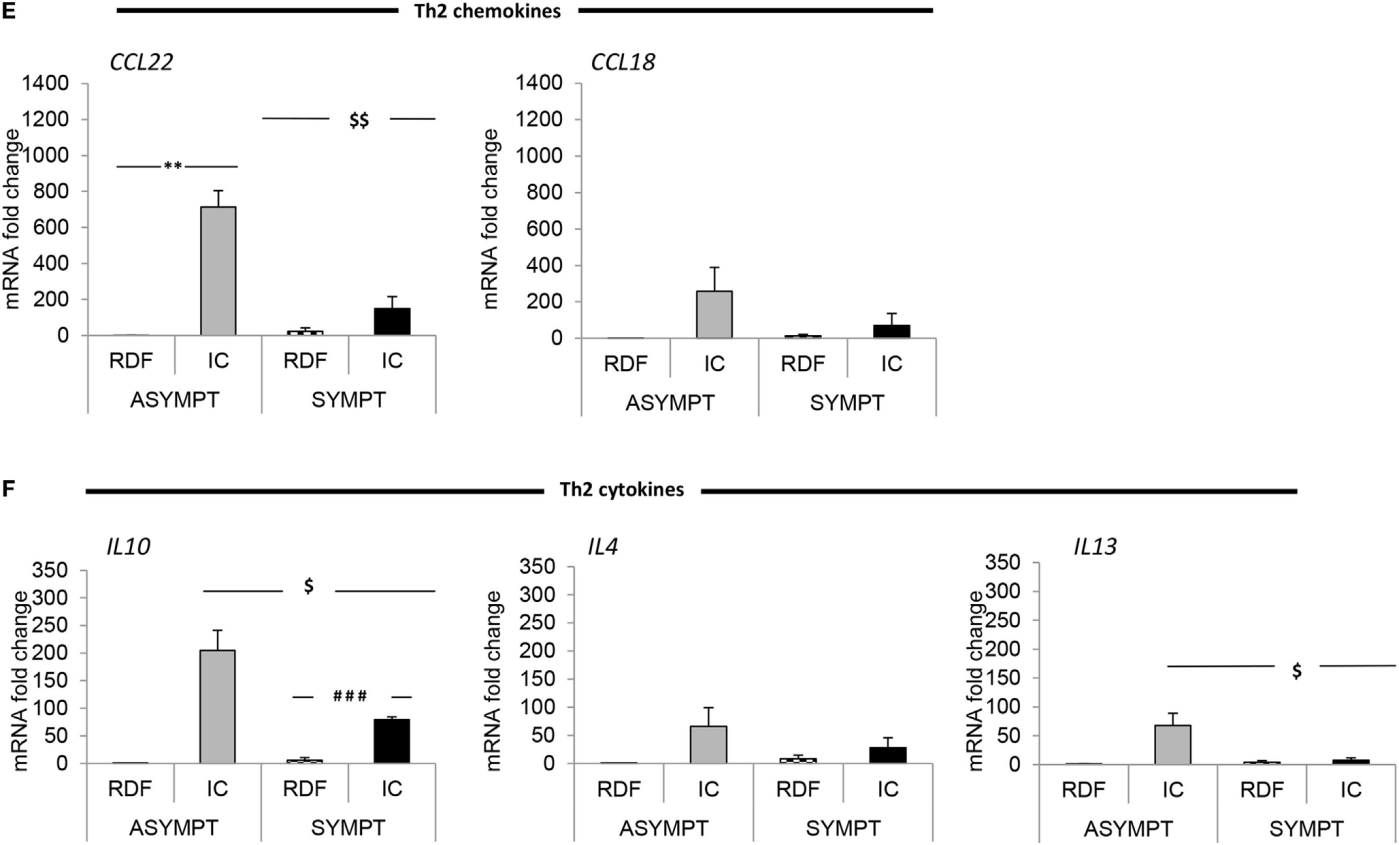

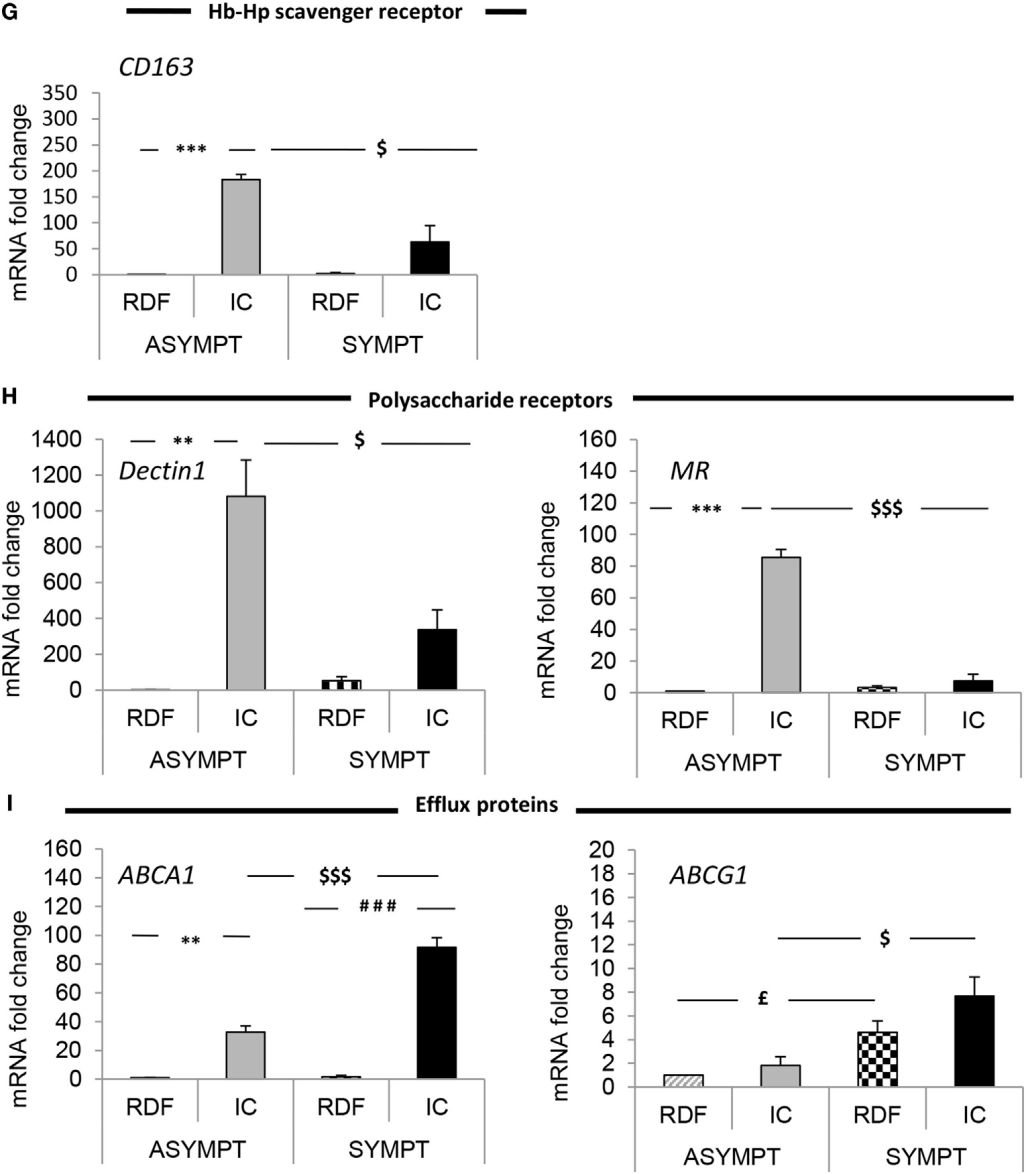
**Macrophage polarization marker mRNA analysis in symptomatic vs. asymptomatic patients**. The panels display mRNA fold change expression of 22 macrophage markers over control (RDF in asymptomatic patients). RT-PCR analysis showed that **(A)** in both asymptomatic and symptomatic plaques low level of the LPS-recognition receptor, CD14 is expressed. In symptomatic plaques the macrophage profile is characterized by increased expression of **(B)** ox-LDL scavenger receptors (CD68, CD36, and SRA-1), Th1-associated **(C)** chemokines (CXCL10 and MCP-1) and **(D)** cytokines (TNFα, IL-1β, IL-6, IL-8, IL-12p40, and IL-12p35) and **(I)** efflux proteins (ABCA1 and ABCG1), compared with asymptomatic patients, suggesting a predominant MΦ1 phenotype among the whole macrophage population. Conversely, asymptomatic lesions are primarily composed of MΦ2-like macrophages, characterized by increased expression of Th2-associated **(E)** chemokines (CCL22 and CCL18) and **(F)** cytokines (IL-10, IL-4, and IL-13), **(G)** Hb–Hp scavenger receptor (CD163) and **(H)** polysaccharide receptors (Dectin 1, MR) compared to symptomatic plaques. Data are mean ± SEM where ^* # £ $^*p* < 0.05, ^** ## ££ $$^*p* < 0.01, ^*** ### £££ $$$^*p* < 0.001 vs. controls, after *T*-test analysis of *N* = 3 independent experiments. Controls legend: * indicates comparison between RDF vs. IC in asymptomatic; ^#^ indicates comparison between RDF vs. IC in symptomatic; ^£^ indicates comparison between RDFs in the two subgroups of patients;^$^ indicates comparison between ICs in the two subgroups of patients.

As shown in Figure 4, CD36 is almost equally expressed in asymptomatic and symptomatic plaque. Similarly, the expression of CD14 was unaltered between asymptomatic and symptomatic plaques. However, the previously described pan-macrophage marker CD68 mRNA level was increased by 7.3-fold (*p* < 0.05) in symptomatic relative to asymptomatic plaques, suggesting that the total number of macrophages is higher in symptomatic lesions.

Indeed, the change in CD68 expression is in keeping with the increased expression of MΦ1 markers and Th1 cytokines namely, TNFα, IL-6, and IL-8 all of which were increased in the IC of symptomatic plaques compared with asymptomatic (by 26.6-fold *p* < 0.01; 10.8-fold, *p* < 0.01; and 8.9-fold, *p* < 0.05, respectively) (Table 1). In addition, there was a trend toward increased expression of CXCL10, MCP-1, and IL-1β expression, although this did not reach significance (Table 3; Figure 4). These data, together with the increase in CD68 expression, suggest that the total number of macrophages is higher in symptomatic plaques and is mainly composed of MΦ1.

**Table 3:** **Differential gene expression for established macrophage markers in symptomatic vs. asymptomatic human carotid plaques**.

Gene		Asymptomatic		Symptomatic		IC Sympt vs. IC Asympt	
Function	Name	RDF	IC	RDF	IC	Fold change	*p*-Value
LPS-recognition receptor	CD14	1	3.36	1.39	3.06	0.91	0.8309
Ox-LDL scavenger receptors	***CD68***	1	***1.94***	1.29	***14.23***	***7.32***	***0.0152***
	CD36	1	157.99	1.01	165.85	1.05	0.8197
	***SRA-1***	1	***99.34***	0.76	***221.13***	***2.23***	***0.0087***
Th1 chemokines	CXCL10	1	196.41	0.99	496.03	2.53	0.3428
	MCP-1	1	48.34	3.37	274.51	5.68	0.2319
Th1 cytokines	**TNFα**	1	***5.64***	0.18	***150.16***	***26.64***	***0.0077***
	IL-1β	1	40.58	4.27	90.40	2.23	0.0877
	***IL-6***	1	***25.13***	0.42	***270.79***	***10.78***	***0.0060***
	***IL-8***	1	***31.06***	0.29	***276.31***	***8.90***	***0.0297***
	IL-12p40	1	53.34	3.70	80.72	1.51	0.5378
	IL-12p35	1	42.55	5.26	96.80	2.28	0.4272
Th2 chemokine	***CCL22***	1	***713.12***	22.66	***148.34***	***−4.81***	***0.0076***
	CCL18	1	258.03	12.72	70.63	***−****3.65*	*0.2731*
Th2 cytokines	***IL-10***	1	***204.66***	5.79	***79.08***	***−3.15***	***0.0273***
	IL-4	1	65.72	8.41	28.32	***−***2.32	0.3812
	***IL-13***	1	***67.88***	3.78	***7.65***	***−8.87***	***0.0484***
Polysaccharide receptors	***Dectin-1***	1	***1081.59***	51.50	***337.56***	***−3.20***	***0.0320***
	***MR***	1	***85.56***	3.21	***7.46***	***−3.74***	***0.0003***
Efflux proteins	***ABCA1***	1	***32.66***	1.64	***91.72***	***2.81***	***0.0019***
	***ABCG1***	1	***1.81***	4.61	**7.68**	***4.23***	***0.0290***
Hb-Hp scavenger receptor	***CD163***	1	***182.92***	2.73	***63.03***	***−3.54***	***0.0222***

*mRNA fold change expression of 22 macrophage markers over control (RDF in asymptomatic patients) analyzed by RT-PCR. A fold change analysis of ICs between the two groups is also reported, together with the relative *p*-value after *T*-test correction of *N* = 3 independent experiments. A value of *p* < 0.05 was considered significant. Genes which were significantly altered are indicated in bold*.

Conversely, MΦ2 markers, MR, CD163, and Dectin-1 mRNA levels were decreased in symptomatic compared to asymptomatic (by 3.7-fold, *p* < 0.001; 3.5-fold, *p* < 0.05, and 3.20, *p* < 0.05, respectively). Furthermore, the expression of Th2 cytokines IL-10 and IL-13 and Th2 chemokine CCL22 were significantly decreased in symptomatic compared with asymptomatic plaques (by 3.2-fold, *p* < 0.05; 8.9-fold, *p* < 0.05, and 4.8-fold, *p* < 0.01, respectively) and again there was a trend toward decreased IL-4 and CCL18 expression (Table 3; Figure 4), suggesting a decrease in MΦ2 macrophages in the progression of human atherosclerosis.

Interestingly, expression of the SR SRA-1 and the efflux proteins, ABCA1 and ABCG1, were also increased in symptomatic compared to asymptomatic (by 2.2-fold, *p* < 0.01; 2.8-fold, *p* < 0.01; and 4.2–fold, *p* < 0.05, respectively). This suggests that the increase in SR and ABC- transporter expression is associated with an MΦ1-like phenotype. In summary, the total number of macrophages is higher in symptomatic lesions, which are mainly composed of MΦ1 and displaying a high expression of Th1 cytokines and SRA-1, ABCA1, and ABCG1 expression. Conversely, although fewer in absolute number of macrophages, asymptomatic lesions are mainly composed of MΦ2 macrophage, characterized by an up-regulation of MR, CD163, and Dectin-1 and Th2 cytokines and chemokines.

**Figure 5:**
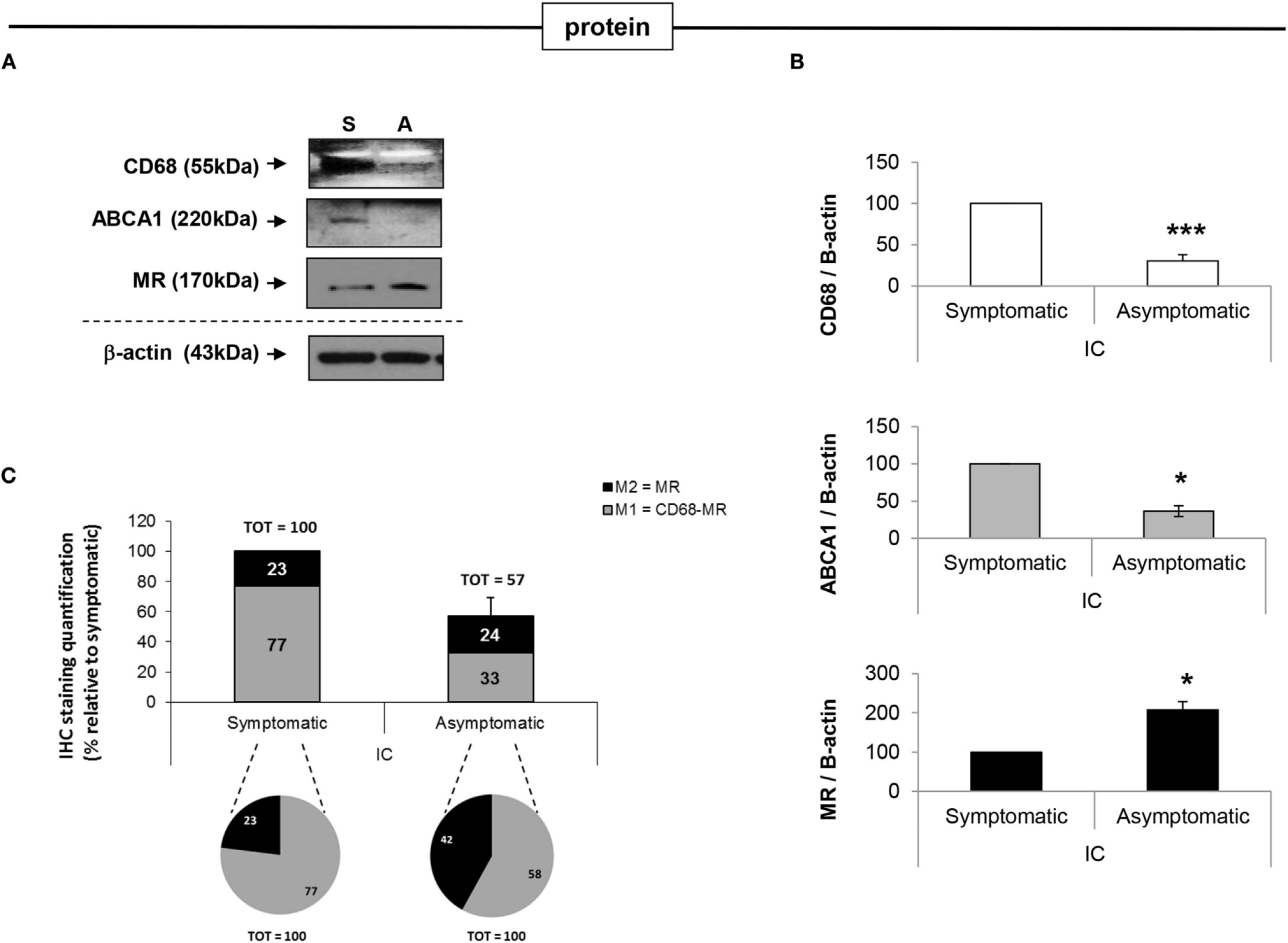
**Analysis of protein expression of MΦ1 and MΦ2 markers in symptomatic vs. asymptomatic patients**. **(A)** Western Blot and **(B)** the relative densitometry analysis, confirm RT-PCR analysis of selected macrophage markers, where ABCA1 is increased and MR is decreased in symptomatic (S) compared with asymptomatic (A) plaques, suggesting a switch from MΦ2 to MΦ1 macrophage thus enriching the pro-inflammatory fraction population, as disease progresses. **(C)** Protein expression was also quantified by IHC analysis to determine the relative distribution of MΦ1 and MΦ2 macrophage populations, within asymptomatic and symptomatic plaques. Quantification of CD68 and MR staining is shown. In symptomatic plaques, MΦ1 macrophages are increased relative to MΦ2. In addition, the absolute number of MΦ1 macrophages is increased in symptomatic compared to asymptomatic plaques. As expected, the percentage of MΦ2 macrophage, relative to MΦ1 cells, is significantly greater in asymptomatic compared to symptomatic patients (pie charts), even though their absolute number between symptomatic and symptomatic lesions is similar (bars). For all the experiments above mentioned, data are mean ± SEM where *p < 0.05, **p < 0.01, ***p < 0.001 vs. symptomatic after *T*-test analysis of *N* = 3 independent experiments.

Western blot analysis of representative macrophage markers altered between asymptomatic and symptomatic patients, namely CD68, MR, and cholesterol efflux ABCA1, confirmed the mRNA expression data. Briefly, CD68 expression in asymptomatic plaques was decreased by 3-fold (*p* < 0.001) compared to symptomatic plaques; MR expression was increased by 2-fold (*p* < 0.05) and ABCA1 was decreased by 2.7-fold (*p* < 0.05) (Figures 5A,B).

To further expand protein analysis, quantification of IHC stained section was used to identify macrophage subpopulations. Using the “Nuclear” and the “Color Deconvolution” algorithms CD68 and MR protein expression was quantified (Figure 5C). The data clearly illustrate that the number of total macrophage in asymptomatic patients is significantly lower than those of symptomatic patients [represented by only 57 ± 12% of the total number of macrophage in the symptomatic group (*p* < 0.05)] (Figure 5C – bars). Although MΦ2 macrophages are relatively abundant in asymptomatic plaques, representing 42 ± 5% of the total macrophage population, this population significantly decreases with progression of disease, where MΦ2 macrophages represent only the 23 ± 3% of the total macrophage population in symptomatic plaques (*p* < 0.05). Furthermore, the relative percentage of MΦ1 is significantly higher in symptomatic plaques, compared to asymptomatic plaques where this population represents 77 ± 5% vs. 58 ± 2% of the total macrophage population in symptomatic and asymptomatic plaques, respectively (Figure 5C). The change in relative abundance of MΦ2 populations as disease progresses is likely due to both an increase in the number of pro-inflammatory macrophages and a direct effect on the anti-inflammatory fraction. These data, based on localization and quantification of MΦ1 and MΦ2 macrophage subpopulations, within both symptomatic and asymptomatic plaques suggest that the MΦ2 macrophage subpopulation may play potential protective role in human atherosclerosis. However, detailed characterization of the macrophage populations warrant further investigation.

## Discussion

Despite atherosclerosis being defined as a chronic inflammatory condition, paired with lipid accumulation, there are no therapies that induce regression of pre-established disease. This is important as most patients present clinically with established disease and the therapeutic goal is to reverse the lesion together with the prevention of the risks associated with the plaque presence, such as plaque rupture and thrombosis. Surgical revascularization is the current intervention employed to treat advanced atherosclerosis. Although clinically endarterectomy is effective, it does not fully address the long-term management of the disease. Therefore, plaque regression is the quintessential target of atherosclerosis intervention. By decreasing plaque size, stenosis can be relieved, removing not only the risks associated with plaque size, rupture, and thrombosis but also reducing the requirement for surgical intervention. There is a general consensus that a stenosis of <70% does not require surgical intervention, as the risks of surgery outweigh the risks presented by stenosis. However, stenosis >70% is deemed to be a greater risk than its surgical removal. Regression of an atherosclerotic lesion into the “<70%” stenosis group in itself would be therapeutically beneficial (9, 44).

Our laboratory has previously shown that dietary administration of CLA induces regression of pre-established atherosclerosis *in vivo* (16) and has identified the monocyte/macrophage as the cellular target through which CLA mediates this profound effect (17, 18). Importantly, we have previously shown that, in a CLA-induced model of atherosclerosis regression, there is enrichment of genes associated with the MΦ2 alternatively activated macrophage phenotype in the murine aorta (19). This is in agreement with other published data that show decreased lipid levels are accompanied by lower plaque lipid content and higher expression of genes for MΦ2 macrophages in the *Reversa* mouse model of regression (36).

Most recently, we have shown that CLA primes human peripheral blood monocytes to adapt an MΦ2 phenotype *in vitro* thus mediating atheroprotection, suggesting that interventional tools capable of altering the macrophage infiltrate toward an anti-inflammatory phenotype may be of therapeutic relevance (20). However, to translate this into a clinical setting, a comprehensive understanding of macrophage populations in human atherosclerotic disease progression is required. Indeed, to date, studies on macrophage populations and polarization in human atherosclerotic disease progression are limited.

To address these points, a study was designed in which atherosclerotic plaques from asymptomatic and symptomatic patients undergoing CEA were recruited. To confirm if this was a robust model of disease progression, initial experiments addressed if it was possible to identify cellular changes between the two patient subgroups and, ultimately, if differential macrophage target gene regulation could be detected.

To establish if the differences between symptomatic and asymptomatic disease could be seen at cytological level, the cellular content of atherosclerotic lesions was first verified using H&E. This provided a useful tool in the identification of lesion composition and orientation, highlighting the differences “between” and “within” lesion types. Indeed, the differences in cellular composition were evident upon gross analysis of the plaques and it was observed that asymptomatic plaques are generally less diseased and present with less evidence of hemorrhaging and stenosis. The characteristic differences seen at a cellular level between symptomatic and asymptomatic plaques were also evident, with increased intimal thickening, cholesterol crystal deposition, foam cells, and lipid accumulation and a larger and more developed lipid core seen in the symptomatic plaque compared to the asymptomatic plaque. Moreover, the results presented here show less cellular activity in the asymptomatic compared with symptomatic plaques. RDF was deemed to an appropriate control for the purpose of these studies. It was used as an internal control within the plaque, displaying minimal lipid deposition, SMCs and fibrous deposits, thus indicating a “relative” absence of atherosclerotic development.

As described above, the strategy provides a good model of disease progression and, therefore, this study was extended to identify macrophage subpopulations in asymptomatic and symptomatic plaques, initially using an immunohistochemical approach.

A major aim was to identify the presence of MΦ2 anti-inflammatory macrophages, and to seek evidence that there is a shift in MΦ2 population associated with disease progression. We have recently shown *in vitro* that increasing MΦ2 subpopulations plays a role in atheroprotection (20). Immunohistochemistry analysis was performed using the CD68 pan-macrophage marker, which identified the total number of macrophages present. MΦ2 macrophages were then discriminated by the staining of MR receptor. It was predicted that MΦ2 staining would be scarce in the symptomatic plaques compared to the asymptomatic plaques. The analysis showed that MΦ2 anti-inflammatory macrophages were mainly located in more distal regions and were found overlying the developing core in the asymptomatic plaques. It was noted that, as the disease progressed, these MΦ2 alternative macrophages were suppressed and MΦ1 pro-inflammatory macrophages became predominant features of the plaque. MΦ1 classically activated macrophages were present in abundance in the developed lipid core of the symptomatic plaque and were rarely found in the intimal regions of the plaque. MΦ2 levels were evidently higher in asymptomatic atherosclerotic plaques, which are ultimately less advanced in their disease progression. Furthermore, in line with our findings, Bouhlel et al. showed that both MΦ1 and MΦ2 macrophages have been identified in human atherosclerotic plaque and that MΦ2 macrophages are present at more stable locations within atherosclerotic plaques (23). However, that study did not compare asymptomatic and symptomatic patients. In addition, a previous study by Lee et al. employed laser micro-dissection to examine macrophage mRNA from stable and ruptured plaques using a transcriptomics approach and found significant differences in the gene expression profiles. Of relevance to our study, where we report increase in cholesterol efflux proteins and inflammatory cytokines in disease progression, they showed that Leptin and FABP4, which both have functions linking lipid metabolism to inflammation, were among the most differentially expressed individual genes in disease progression (10). Furthermore, in a separate study, transcriptomics and immunohistochemistry was employed to analyze macrophage subset dynamics in successive stages of atherosclerosis. In keeping with our data, they also showed that MΦ1 and MΦ2 macrophage populations are present throughout disease development and that MΦ1 macrophages are preferentially linked to plaque progression (42).

In this study, we further extended our immunohistochemistry approach to perform nuclear analysis, used to quantify the number of CD68 and MR expressing cells. This novel strategy used an algorithm, “V9 Aperio Nuclear Algorithm,” to determine levels of a target protein in human atherosclerotic plaques, providing an accurate and compelling result. This tool allowed quantification of the percentage of MΦ2 macrophage relative to MΦ1 macrophage in both asymptomatic and symptomatic plaques. “Aperio” digital scanning and quantification is a novel methodology and, for the first time, these types of algorithms have been used to quantify macrophage subsets in human atherosclerotic tissue.

As expected, the proportion of MΦ2 macrophages relative to MΦ1 was much greater in the asymptomatic plaques’ developing core compared to symptomatic. Interestingly, based on unique localization and differential expression of MR, substantial evidence confirmed the presence of at least two macrophage subpopulations within atherosclerotic lesions, as well as strong indication that there is a relative loss of MΦ2 and a proportional increase in MΦ1 macrophages in symptomatic atherosclerotic lesion development.

Finally, characterization of ABCA1 and MR expression confirmed the presence of MΦ1 and MΦ2 subpopulations within lesions, at both mRNA and protein levels. Specifically, MΦ1 were characterized by high levels of Th1 cytokines and chemokines, ox-LDL SRs and efflux proteins and low levels of Th2 cytokines and chemokines, polysaccharide receptors and Hb-Hp SR, while the inverse was defined for MΦ2. Our novel data on expression of MΦ1 and MΦ2 markers in human plaques are summarized in Table 4. It has been suggested that there may be an additional “resident macrophage” subpopulation present alongside MΦ1 and MΦ2 macrophages (45) and in future studies, a specific marker for this unknown population should be used to determine its specific localization and role in atherosclerotic progression. It is important to note that this study employed analysis of whole plaque specimens. To definitively characterize the macrophage response in atherosclerotic disease progression isolation of macrophages from harvested samples followed by identification of their transcriptome and proteome should be performed in a larger population.

**Table 4:** **Macrophage profiling in asymptomatic and symptomatic atherosclerotic patients**.

Macrophage Profiling				
	Gene		Level of expression	
	Function LPS-recognition receptor	Name CD14	Asymptomatic Low	Symptomatic Low
MΦ1-like phenotype	Ox-LDL scavenger receptors	CD68 CD36 SRA-1	Low	High
	Efflux proteins	ABCA1 ABCG1	Low	High
	Th1 chemokines	CXCL10 MCP-1	Low	High
	Th1 cytokines	TNFα IL-1β IL-6 IL-8 IL-12p40 IL-12p35	Low	High
MΦ2-like phenotype	Th2 chemokine	CCL22 CCL18	High	Low
	Th2 cytokines	IL-10 IL-4 IL-13	High	Low
	Polysaccharide receptors	Dectin-1 MR	High	Low
	Hb–Hp scavenger receptor	CD163	High	Low

*Summary of macrophage profiling based on differential mRNA differential expression of the 22 macrophage markers between symptomatic and asymptomatic atherosclerotic plaques. In asymptomatic patients, the macrophage displays an MΦ2-like phenotype, displaying high levels of Th2 cytokines and chemokines, polysaccharide receptors and the Hb–Hp scavenger receptor. Conversely, in symptomatic patients, macrophages display an MΦ1-like phenotype, characterized by high levels of Th1 cytokines and chemokines, ox-LDL scavenger receptors, and efflux proteins, associated with atherosclerotic disease progression*.

Macrophages have previously been characterized as heterogeneous cell populations (23, 24). In work by Chinetti-Gbaguidi et al. and Bouhlel et al., it has been proposed that PPARγ activation may effectively prime the differentiation of primary human macrophage into MΦ2 phenotype, generating anti-inflammatory and atheroprotective populations. More recently, it has been shown that MΦ1 macrophage content of atherosclerotic plaques is associated with clinical incidence of ischemic stroke and increased inflammation or fibrinolysis (32). In the context of atherosclerosis, it has been shown that there is an MΦ2 to MΦ1 switch during plaque progression, likely due to a conversion of cells already present in the lesion, suggesting that interventional tools, able to revert the macrophage infiltrate toward the MΦ2 phenotype, may exert an atheroprotective action (32).

We have shown that alteration in macrophage populations in human disease drives atherosclerosis progression. Therefore, future work, aimed to better understand the mechanism through which MΦ1-MΦ2 shift is induced needs to be performed. The ultimate aim of this investigatory field is to find novel therapeutic targets in the treatment of human atherosclerosis, as an alternative type of intervention to the surgical, procedure. Therefore, the experimental model described above will also facilitate identification of shift in macrophage populations in response to current and novel therapeutics.

## Author Contributions

MdG participated in the design of the study, carried out the experiments and prepared the manuscript. DC participated in the design of the study and facilitated analysis. MB performed surgical carotid endarterectomy in patients. OB conceived and designed the study and prepared the manuscript. All authors read and approved the final manuscript.

## Conflict of Interest Statement

The authors declare that the research was conducted in the absence of any commercial or financial relationships that could be construed as a potential conflict of interest.
