# Supplementary material for: M1- and M2-Type Macrophage Responses Are Predictive of Adverse Outcomes in Human Atherosclerosis
Source: Front Immunol. 2016 Jul 19;7:275. doi: 10.3389/fimmu.2016.00275 (PMC4949256; doi:10.3389/fimmu.2016.00275)
Supplement: Supplementary file 1 [file Presentation_1.PDF]

Supplementary Material

Comprehensive analysis of macrophage type-1 and type-2 populations in human atherosclerosis

Monica de Gaetano[1], Mary Barry[2] and Orina Belton[1*]

*** Correspondence:** Dr Orina Belton: orina.belton@ucd.ie

1. Supplementary Figures and Tables

1.1 Supplementary Figures

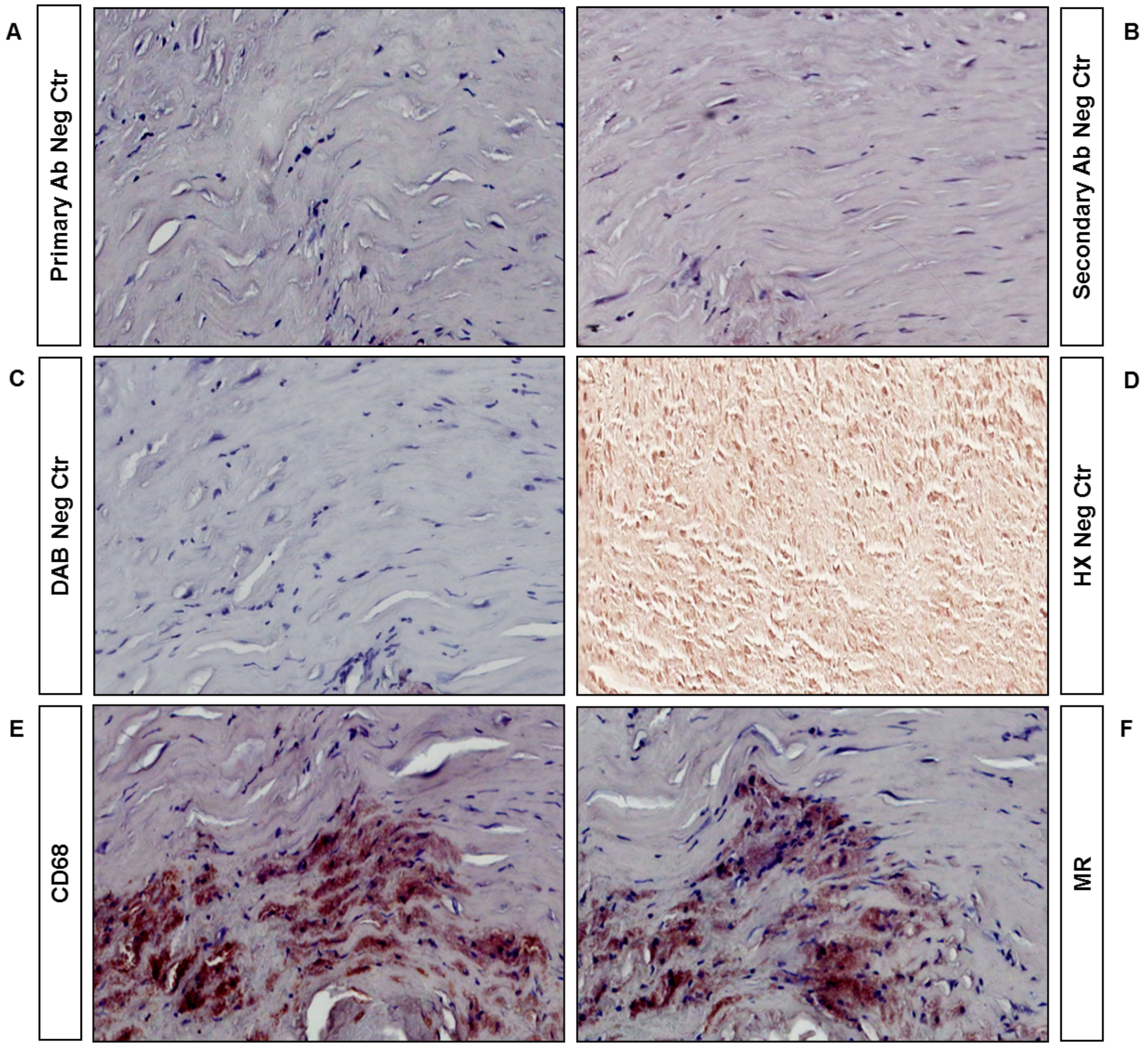

Supplementary Figure 1 - IHC negative controls - The primary and secondary antibody negative controls highlight the quality of the staining, in which DAB doesn't bind tissue in the absence of the primary **(A)** or the secondary antibodies **(B)**. *Vice versa*, primary and secondary antibodies are not possible to be visualized in the absence of DAB **(C)**. In the absence of HX **(D)**, instead, it is not possible to visualize nuclei (elsewhere displayed in blue). CD68 **(E)** and MR **(F)** positive staining panels are indeed reported to show the specificity of action of two different primary antibodies in the presence of both DAB and HX.

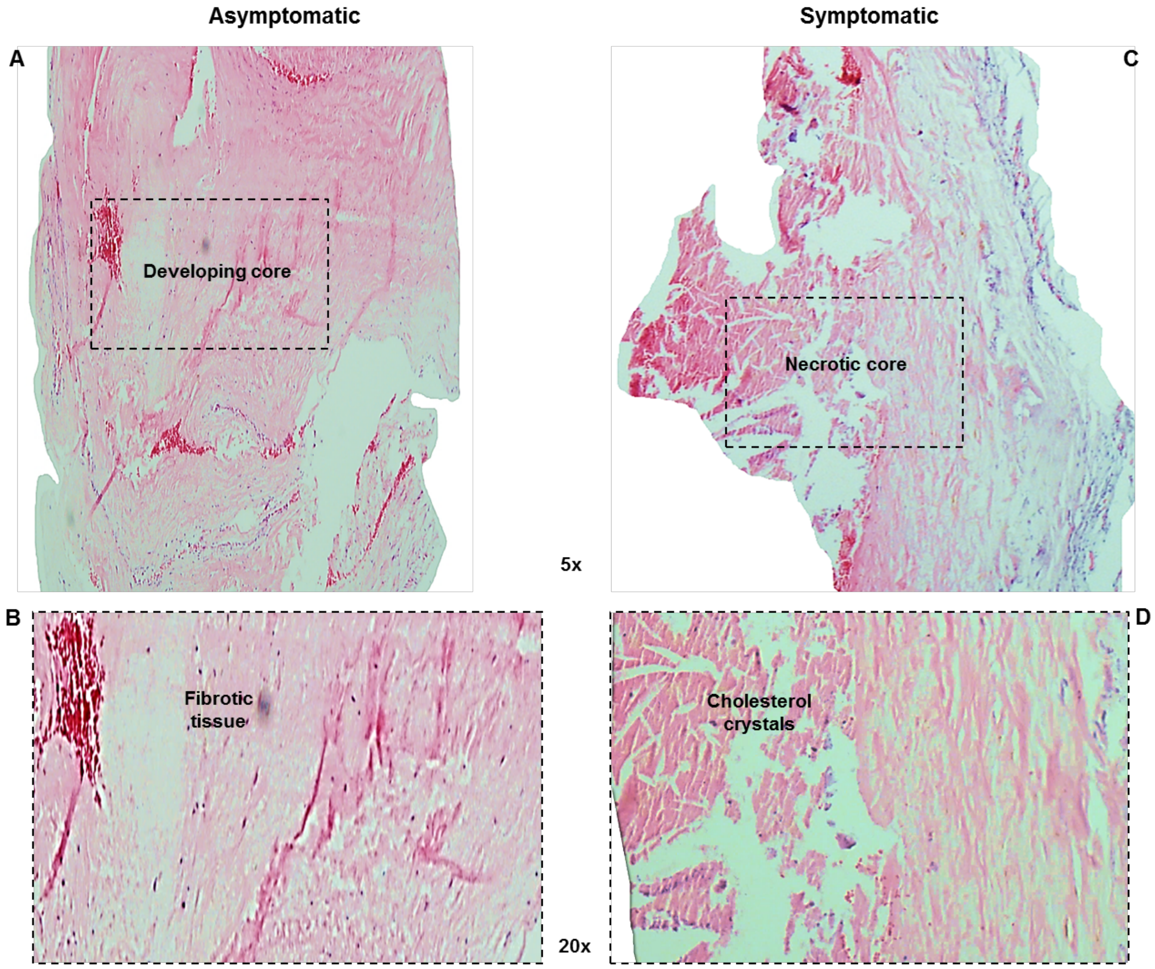

Figure S2 - H&E comparison of core formation between asymptomatic *vs* symptomatic patients. The images displayed here show the staining of 6 um tissues from IC sections from asymptomatic and symptomatic patients. **(A, B)** These two panels are representative of the development of atheroma in an asymptomatic plaque, characterized by fibrotic component. **(C, D)** These two panels clearly indicate the necrotic nature of a developed core of a symptomatic plaque, with its characteristic "fatty-streak" cholesterol deposits. Pictures were captured using TLM (5 and 20x magnifications). Representative images of N=3 independent staining are displayed here.

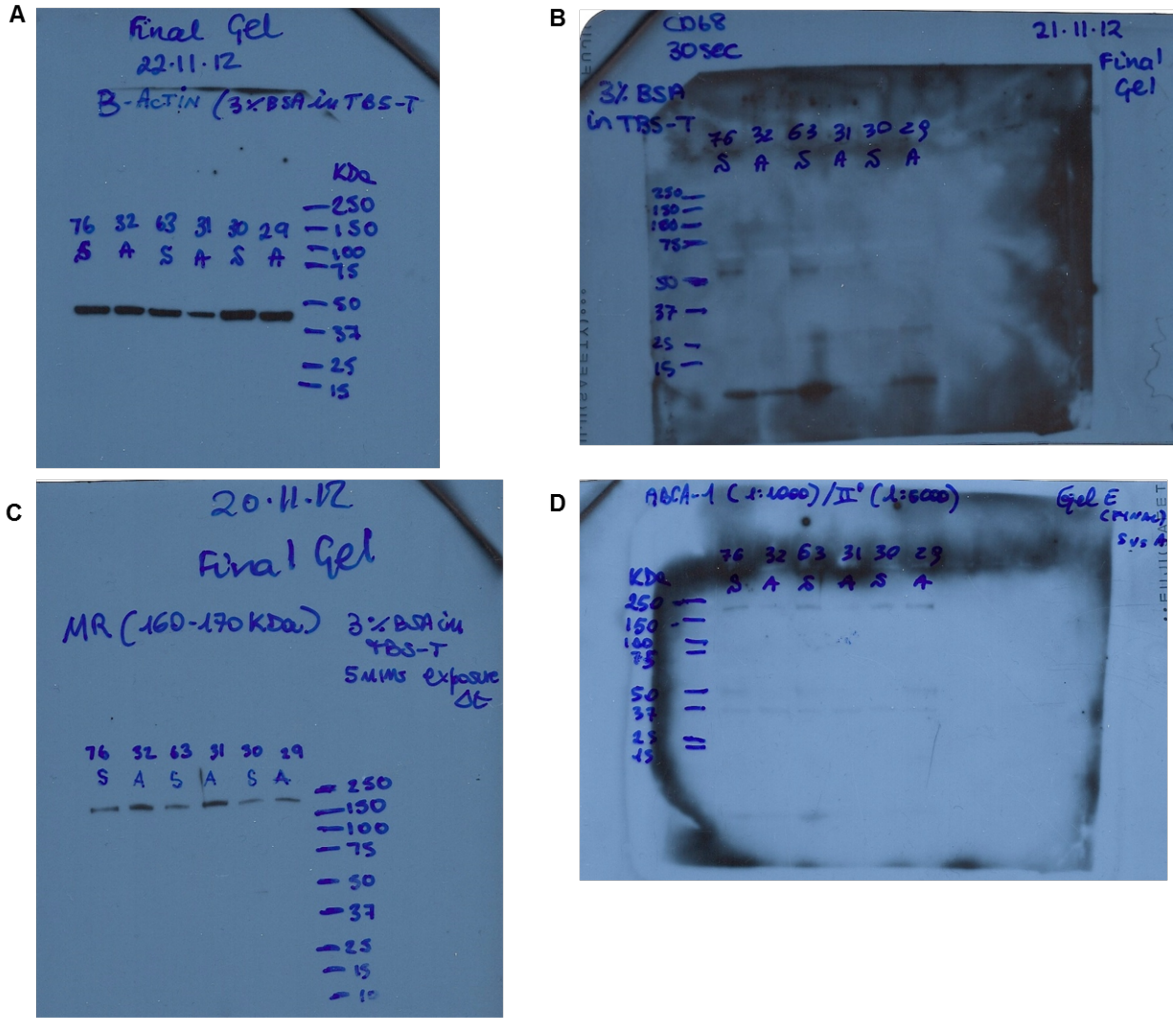

Figure S3 - Western blots analysis of symptomatic *vs* asymptomatic patients. (A) Mouse-monoclonal anti-human β-actin (1:1000, 43 kDa); **(B)** goat-polyclonal anti-human CD68 (1:500, 55 KDa); rabbit-polyclonal anti-human **(C)** MR (1:500, 170 kDa) and **(D)** ABCA1 (1:1000, 220 kDa) original blot are here displayed.

1.2 Supplementary Tables

Supplementary Table 1 - Steps of the 9 hours cycle automated processing of plaque samples. After dissection of the whole specimen into four sections (IC, EC, CC, RDF), wax embedded tissue blocks were processed through increasing graded ethanol series, followed by several passages in chloroform.

Solvent	Duration
**Ethanol (50%)**	45 min
**Ethanol (70%)**	45 min
**Ethanol (95%)**	45 min
**Ethanol (100%)**	45 min
**Ethanol (100%)**	45 min
**Ethanol (100%)**	45 min
**Ethanol (50%) + Chloroform (50%)**	45 min
**Chloroform (100%)**	45 min
**Chloroform (100%)**	45 min
**Chloroform (100%)**	45 min

Supplementary Table 2 - Deparaffinization and rehydration steps of plaques processing. Prior to H&E or immunohistochemical staining, slides were deparaffinized in xylene, in order to remove the wax used to embed the tissue. Subsequently, tissues were rehydrated through a decreasing graded alcohol series, as here documented.

**Solvent**	**Duration**
**Xylene (100%)**	10 min
**Xylene (100%)**	10 min
**Ethanol (100%)**	10 min
**Ethanol (90%)**	9 min
**Ethanol (70%)**	8 min
**Ethanol (50%)**	7 min
**Ethanol (30%)**	5 min
**Pure water**	3 min

Supplementary Table 3 - Dehydration steps after immunostaining. Following H&E or immunohistochemical staining, tissues were dehydrated through an increasing graded alcohol series. Subsequently, two xylene washing steps were performed, as here reported.

**Solution**	**Duration**
**Pure water**	3mins
**Ethanol (30%)**	5mins
**Ethanol (50%)**	7mins
**Ethanol (70%)**	8mins
**Ethanol (90%)**	9mins
**Ethanol (100%)**	10mins
**Xylene (100%)**	10mins
**Xylene (100%)**	10mins

Supplementary Table 4 - Human Syber Green primer sequences. Relative gene expression quantification by RT-PCR was performed on an ABI Prism 7900HT Sequence Detection System, (Applied Biosystems Inc., UK). MR, SRA1 and ABCG1 expression were examined using specific Taqman assays (Applied Biosystems Inc., UK), whilst, TNFα, IL-1β, IL-6, IL-8, IL-12p40, IL12p35, CXCL10, MCP-1, Dectin1, CCL22, CCL18, IL10, IL4, IL13, ABCA1, CD14, CD36, CD68 and CD163 target genes were measured using specific Syber green assays (Eurofins, MWG Operon, Germany). Ct values were then normalised to 18s ribosomal RNA. In this table, sense (forward primer 5'-3') and antisense (reverse primer 5'-3') Syber Green primers' sequences are displayed for all the amplification targets.

Gene	Sequence	
ABCA1	Sense	GCAGCAGAGCGAGTACTTCGTT
	Antisense	CAAGACTATGCAGCAATGTTTTTGT
CD36	Sense	TGTAACCCAGGACGCTGAGG
	Antisense	GAAGGTTCGAAGATGGCACC
CD14	Sense	CGCTCCGAGATGCATGTG
	Antisense	AACGACAGATTGAGGGAGTTCAG
CD68	Sense	GCTACATGGCGGTGGAGTACAA
	Antisense	ATGATGAGAGGCAGCAAGATGG
CD163	Sense	CGAGTTAACGCCAGTAAGG
	Antisense	GAACATGTCACGCCAGC
TNFα	Sense	CTCGAACCCCGAGTGACAA
	Antisense	GCTGCCCCTCAGCTTGAG
IL1β	Sense	CCA CGG CCA CAT TTG GTT
	Antisense	AGG GAA GCG GTT GCT CAT C
IL6	Sense	AGCCGCCCCACACAGA
	Antisense	CCGTCGAGGATGTACCGAAT
IL8	Sense	AAGGAACCATCTCACTGTGTGTAAAC
	Antisense	ATCAGGAAGGCTGCCAAGAG
IL12p40	Sense	CGGTCATCTGCCGCAAA
	Antisense	TGCCCATTCGCTCCAAGA
IL12p35	Sense	CTCCTGGACCACCTCAGTTTG
	Antisense	GGTGAAGGCATGGGAACATT
CXCL10	Sense	TCGAAGGCCATCAAGAATTT
	Antisense	GCTCCCCTCTGGTTTTAAGG
MCP1	Sense	CCCCAGTCACCTGCTGTTAT
	Antisense	TGGAATCCTGAACCCACTTC
Dectin1	Sense	GGGAATCCTATGCTTGGTAAT
	Antisense	TGGAGATGGGTTTTCTTGGG
CCL22	Sense	GTTGTCCTCGTCCTCCTTGC
	Antisense	GGAGTCTGAGGTCCAGTAGAAGTG
CCL18	Sense	TCTATACCTCCTGGCAGATTC
	Antisense	TTTCTGGACCCACTTCTTATTG
IL4	Sense	ACTTTGAACAGCCTCACAGAG
	Antisense	TTGGAGGCAGCAAAGATGTC
IL10	Sense	GCCTAACATGCTTCGAGATC
	Antisense	TGATGTCTGGGTCTTGGTTC
IL13	Sense	TGA GGA GCT GGT CAA CAT CA
	Antisense	CAGGTTGATGCTCCATACCAT
